# Deep Efficient Data Association for Multi-Object Tracking: Augmented with SSIM-Based Ambiguity Elimination

**DOI:** 10.3390/jimaging10070171

**Published:** 2024-07-16

**Authors:** Aswathy Prasannakumar, Deepak Mishra

**Affiliations:** Department of Avionics, Indian Institute of Space Science and Technology, Trivandrum 695547, Kerala, India; deepak.mishra@iist.ac.in

**Keywords:** multiple object tracking, object detection, data association, feature association matrix, structural similarity index metric

## Abstract

Recently, to address the multiple object tracking (MOT) problem, we harnessed the power of deep learning-based methods. The tracking-by-detection approach to multiple object tracking (MOT) involves two primary steps: object detection and data association. In the first step, objects of interest are detected in each frame of a video. The second step establishes the correspondence between these detected objects across different frames to track their trajectories. This paper proposes an efficient and unified data association method that utilizes a deep feature association network (deepFAN) to learn the associations. Additionally, the Structural Similarity Index Metric (SSIM) is employed to address uncertainties in the data association, complementing the deep feature association network. These combined association computations effectively link the current detections with the previous tracks, enhancing the overall tracking performance. To evaluate the efficiency of the proposed MOT framework, we conducted a comprehensive analysis of the popular MOT datasets, such as the MOT challenge and UA-DETRAC. The results showed that our technique performed substantially better than the current state-of-the-art methods in terms of standard MOT metrics.

## 1. Introduction

Vision-based multiple object tracking (MOT) is a longstanding research problem with broad applications in computer vision such as intelligent surveillance systems, robotics, human–computer interaction, medical image processing, and autonomous driving. The MOT algorithm provides a robust framework for real-time monitoring and analysis of multiple moving objects, enabling accurate tracking and prediction of their movements in various dynamic scenarios. The tracking-by-detection paradigm is widely recognized as the most effective approach to multiple object tracking (MOT). It involves utilizing an efficient object-detection algorithm to identify objects within each frame of a video sequence. Subsequently, a data association algorithm is employed to establish associations between detections across frames, thereby creating object trajectories [1,2,3]. Although various approaches have been presented to handle the problem, MOT is still a challenging research area due to factors like object occlusions, the varying number of objects per frame, abrupt appearance changes, etc.

The state-of-the-art MOT concepts have become more potent with recent advances in deep learning. MOT treats object detection and data association as two independent tasks. Modern advancements in deep learning have led to the development of highly effective off-the-shelf object detectors capable of accurately detecting various objects in complex scenes [4,5,6,7,8,9,10]. Once object detections are obtained in each frame, the subsequent task of data association focuses on linking these detections across consecutive frames to establish object trajectories over time. Data association remains a challenging task in its own right and has not fully leveraged the advancements in deep learning. The standard process of data association typically involves extracting representative features from individual detections and then matching them with existing object trajectories using specified similarity metrics. Deep learning networks offer enhanced capability for learning robust feature representations of objects. In our study, we employed a modified VGGNet deep network for hierarchical feature learning and extraction from all detected objects. This deep feature extractor allowed us to capture distinctions and variations in object appearances, improving the accuracy and reliability of object associations over time.

After extracting features for detections in each frame, the next task is to associate these detections with previously tracked objects. This association involves comparing the extracted features of detections with those of existing object trajectories to find the most suitable matches. Detections with the highest similarity scores are linked to their corresponding trajectories. Our research mainly focuses on enhancing the data association task within multiple object tracking (MOT) frameworks. We propose an efficient association framework that integrates a deep feature association network (deepFAN) and the Structural Similarity Index Metric (SSIM) [11,12,13]. This framework jointly calculates association scores for object detection–target pairs. The deepFAN learns the complex feature association function to encode the association score between detections and tracked targets, while the SSIM handles uncertainties in association by comparing its feature similarities. By combining the deep learning capabilities of the deep feature association network with SSIM, our approach aims to improve the accuracy and reliability of object associations across frames.

Traditionally, affinities in multiple object tracking (MOT) are calculated by exhaustively evaluating all possible pairs of detection and target features. In contrast, our proposed MOT framework integrates a neighborhood detection estimation (NDE) module to refine this process, selecting a more reliable subset of detection–target pairs. The NDE module enhances efficiency by focusing on nearby or contextually relevant detections rather than evaluating every possible permutation. This filtering step improves the quality of associations by prioritizing those with a higher likelihood of accuracy. In our framework, the deep feature association network (deepFAN) and the Structural Similarity Index Metric (SSIM) jointly determine the association score for these refined pairs. Furthermore, the training method we employed for the deep feature association network (deepFAN) enables efficient object association across multiple frames in a video sequence, ensuring reliable trajectory tracking. During training, the network is exposed to input frame pairs that are not necessarily consecutive. This strategy proves beneficial by allowing the framework to link objects across non-adjacent frames. This capability reduces instances of identity switches and fragmented object trajectories. By integrating the NDE module and optimizing deepFAN training, our MOT framework enhances tracking accuracy while maintaining computational efficiency.

This study presents a systematic approach to estimating an efficient association matrix for multiple object tracking (MOT), which effectively summarizes the correspondence between current frame detections and previously estimated target trajectories. Leveraging the capabilities of deep learning architectures, our proposed framework integrates innovative components aimed at enhancing MOT performance. Key components of our approach include the following:

- The proposed data association framework employs the deep feature association network (deepFAN) along with the Structural Similarity Index Metric (SSIM) to estimate an efficient association matrix. This combination improves the robustness of object associations by leveraging deep learning for feature extraction and similarity evaluation.
- In the proposed data association framework, a neighborhood-detection-estimation (NDE) scheme is introduced to select a reliable subset of detection–target pairs. This neighborhood detection estimation, along with post-processing steps within the deep feature association network, contributes to enhancing the computational efficiency. Experimental evaluations highlight that the proposed approach minimizes incorrect associations, thereby improving overall tracking performance.
- A specialized training strategy is developed for the deep feature association network (deepFAN), allowing the network to utilize non-consecutive frame pairs for the effective learning of the data association function. This method improves the overall ability of the network to link objects across frames, thereby reducing identity switches and fragmented trajectories.

We validated the effectiveness of each component through ablative experiments on the MOT validation dataset. Additionally, comprehensive analyses on the benchmark datasets, including MOT15, MOT17, MOT20, and UA-DETRAC, demonstrated that our method achieved competitive and state-of-the-art results across various MOT evaluation metrics. The MOT metric scores for identity switches, fragmentation, and false negatives were reduced, indicating the reduction in the wrong association among detection target pairs.

The rest of the article is organized as follows: Section 2 reviews the existing literature on multiple object tracking (MOT). Section 3 details the methodology employed in the online multiple object tracking framework. In Section 4, we present the experimental findings and comparative results and discuss them in depth. Finally, Section 5 concludes the study with a summary of the findings and suggestions for future research directions.

## 2. Related Works

To obtain a comprehensive overview of the multiple object tracking (MOT) problem, we refer to foundational studies [14,15,16]. Within MOT frameworks, the tracking-by-detection approach is the most commonly utilized method [1,2,3]. The effectiveness of this approach relies heavily on the quality of object detections and the accuracy of trajectory estimation. The recent advancements in deep learning have significantly improved object detectors [4,5,6,7,8,9,10], leading to better object detection performance and, consequently, enhancing the overall efficiency of the MOT framework.

This discussion will focus specifically on data association approaches used for trajectory estimation in MOT. An essential step for any data association method is computing representative features of the detections in each frame. Several approaches exist for determining representation models, including appearance-based [17,18,19], motion-based [20,21,22], and composite models [23,24]. For MOT frameworks, deep learning-based feature extraction methods provide robust and discriminative representation models for object detections, which significantly boost tracking performance. Typically, pre-trained classification or object detection models are employed for feature extraction in tracking tasks [25,26,27,28,29]. In particular, ShiJie et al. [30] proposed a deep affinity network that jointly learns representational features and their affinities with targets. Our proposed MOT framework adopts the feature extraction model utilized in [30].

The study by Emami et al. [31] views data association as a multidimensional assignment problem and consolidates many popular learning algorithms employed for MOT data association. Researchers have explored various methodologies, including non-probabilistic algorithms, probabilistic graphical models, Markov Chain Monte Carlo (MCMC), and deep learning techniques to solve the data association problem. Among non-probabilistic approaches, the Greedy Randomized Adaptive Search Procedure (GRASP) is frequently used for multi-sensor multi-object tracking [32]. In probabilistic graphical models, common techniques include network optimization [33,34], conditional random fields [35,36,37], and belief propagation [38,39]. Additionally, MCMC has been a valuable tool for data association in multiple object tracking [40,41].

In recent years, there have been numerous successful attempts to formulate data association in MOT using deep learning methods. The Deep Affinity Network (DAN) proposed by ShiJie et al. [30] represents an end-to-end trainable deep network that jointly learns feature modeling and association estimation. Similarly, FAMNet [42] leverages deep networks for both feature extraction and association estimation. Yihong et al. introduced the Deep Hungarian Network (DHN) [43], which predicts associations from a cost matrix derived from detections and targets. The Dual-Matching Attention Network (DMAN) [44] employs spatial and temporal attention mechanisms to predict and refine association assignments. The integration of deep models such as Recurrent Neural Networks (RNNs) [45], autoencoders [46], and Generative Adversarial Networks (GANs) [47] into the data association problem has led to significant improvements in MOT performance.

This work presents a systematic approach to data association within the MOT framework that harnesses the power of deep learning models. The proposed MOT algorithm for track association enhances both computational efficiency and tracking accuracy. By leveraging the potential of deep learning models, our method aims to address the complexities and challenges associated with data association in MOT, ensuring more reliable and effective tracking outcomes.

## 3. Methodology: Online Multiple Object Tracking Framework

In the tracking-by-detection paradigm of MOT, the process involves two distinct modules: the object detector and the object tracker. The object detector initially identifies target objects by generating bounding boxes in each video frame. From these bounding boxes, we compute the center locations of objects, $C_{D}^{f}$ for frame $I^{f}$. Our proposed MOT framework is designed to seamlessly integrate with existing multi-object detection methods. We evaluated our approach across various online challenges in multiple object tracking, where state-of-the-art object detectors provide the initial object detections. Specifically, we utilized detections from prominent MOT challenges such as MOT15, MOT17, MOT20 [48,49], and UA-DETRAC [50,51]. Each challenge provides video sequences annotated with detections generated by specific detectors designated for the challenge.

**Figure 1:**
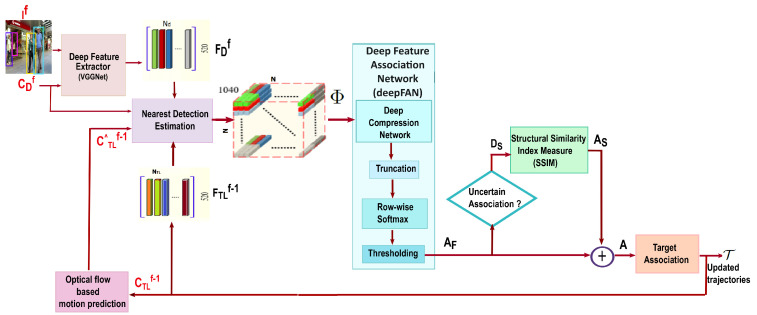
Representation of the proposed MOT framework: Inputs to the framework are the current frame $I^{f}$ and the centers of the object detections $C_{D}^{f}$; the output is the estimated trajectory of the all the targets for frames till $I^{f}$. The two main proposals in the framework are neighborhood detection estimation (NDE) and data association framework with deepFAN and SSIM. The detection feature matrix $F_{D}^{f}$ obtained from the deep feature extractor and matrix with existing targets feature vector $F_{TL}^{f-1}$ are given to NDE to find reliable detection-target pairs and encode them on 3D tensor Φ. The input to the data association network is Φ, and the output is the association matrix, $A\in R^{N_{TL}\times N_{d}}$; the scalar scores, $A_{\left(i,j\right)}$ represents the association score between the $j^{th}$ detection and $i^{th}$ target. Trajectory list T updated using association matrix A for $I^{f}$.

The block diagram representation of the proposed MOT framework is shown in Figure 1. One of the significant components of the proposed MOT framework is a deep feature extractor using a modified VGGNet architecture. The architecture of the feature extractor employed in the proposed framework is based on the state-of-the-art MOT framework described in Reference [30]. The system is expertly developed to efficiently extract comprehensive and compact features from the input object detections. The pretrained VGGNet architecture is fine-tuned within the context of multiple object tracking using training sequences of the MOT benchmark. As shown in Figure 1, the representative feature of each object is obtained by passing the current video frame $I^{f}$ and object centers $C_{D}^{f}$ through the deep feature extractor. For each object detection, a 520-dimensional feature vector is obtained. We refer to [30] for the architectural details of the modified VGGNet feature extractor.

Let $D^{f}={\left\{d_{i}^{f}\right\}}_{i=1}^{N_{d}}$ represent the set of detections given in frame $I^{f}$, where $N_{d}$ is the number of available detections. We acquire a detection feature matrix, $F_{D}^{f}$∈$R^{520\times N_{d}}$, by accumulating the 520-dimensional feature vector for $N_{d}$ detections for each input frame $I^{f}$. This detection feature matrix $F_{D}^{f}$ is then made available for the data association task.

### 3.1. Data Association Methodology

In this section, we extend our discussion on the proposed data association framework that incorporates a deep feature association network and the Structural Similarity Index Metric along with neighborhood detection estimation to tackle the problem. The association algorithm identifies a correspondence between the object detections in the current frame and existing trajectories from the previous frames. This involves comparing the extracted features of detections with those of existing object trajectories to find the most suitable matches. Detections with the highest similarity scores are linked to their corresponding trajectories. Here, we employed a deep feature association network (deepFAN) that consists of a pre-trained CNN-based compression network and an image similarity metric, the Structural Similarity Index Metric (SSIM), to estimate the data association efficiently. This part of the MOT framework computes a feature association matrix A, which encodes the pairwise similarities of the detections and the pre-existing targets.

#### 3.1.1. Neighborhood Detection Estimation

Generally, the data association matrix models a global relationship between all the detections in the current frame and the tracked targets from the previous frames. In the proposed method, instead of considering all the combinations, only the reliable detection–target pairs are chosen for the association task. The neighborhood detection estimation methods are employed to identify those detection–target pairs. This method is based on the assumption that the objects are in a smooth motion, i.e., the location of the objects did not drift drastically in subsequent video frames. Therefore, we have to consider only the detections in the neighborhood area of the targets for the data association.

Let $F_{TL}^{f-1}$∈$R^{520\times N_{TL}}$ represent the set of target feature vectors in the previous frame $I^{f-1}$, including the feature vectors of tracked and lost targets.

(1) $$\begin{array}{c} F_{TL}^{f-1}=\left\{F_{T}^{f-1},F_{L}^{f-1}\right\},\\ \mathrm{where}\;F_{T}^{f-1}\in R^{520\times N_{T}},\;F_{L}^{f-1}\in R^{520\times N_{L}},\\ N_{TL}=N_{T}+N_{L}. \end{array}$$

In Equation (1), $F_{T}^{f-1}$ and $F_{L}^{f-1}$ are the feature matrices that consist of the feature vectors of the tracked and lost targets from the previous frame $I^{f-1}$ and $N_{T}$ and $N_{L}$ are the number of active tracked and lost targets.

The neighborhood-detection-estimation algorithm simply relies on the distance between the centers of the detection and target feature vectors. In order to find the distance, we need to define a distance metric. Here, we are adopting the Euclidean distance with an additional scaling factor. Let $C_{D}$ = $\left\{C_{D_{x}},C_{D_{y}}\right\}$ and $C_{TL}$ = $\left\{C_{{TL}_{x}},C_{{TL}_{y}}\right\}$ be the centers of the detections and targets. The scaled Euclidean distance $E_{s}$ between a detection and a target with centers $\left(c_{D_{x}},c_{D_{y}}\right)$ and $\left(c_{t_{x}},c_{t_{y}}\right)$ is defined as

(2) $$E_{s}=\frac{\sqrt{{\left(c_{D_{x}}-c_{t_{x}}\right)}^{2}+{\left(c_{D_{y}}-c_{t_{y}}\right)}^{2}}}{\sqrt{I_{x}^{2}+I_{y}^{2}}},$$

where ($I_{x}$, $I_{y}$) represents the size of the video frame.

**Optical flow-based motion prediction:** From the object detection bounding boxes, we can determine the center locations of all the detections in the image frame, $C_{D}^{f}$. Further, we have the locations of the tracked and lost targets $C_{TL}^{f-1}$ in the frame $I^{f-1}$ as feedback information from the previous target trajectories. The possible locations of these targets in the present frame $I^{f}$, ${\hat{C}}_{TL}^{f}$, are estimated using the optical flow motion model. Specifically, knowing the target center in $I^{f-1}$, $c_{t}^{f-1}$ = $\left\{c_{t_{x}}^{f-1},c_{t_{y}}^{f-1}\right\}$, we compute its corresponding location ${\hat{c}}_{t}^{f}$ in the following frame ($I^{f})$ using the Lucas–Kanade optical flow method with pyramids [52].

(3) $${\hat{c}}_{t}^{f}=c_{t}^{f-1}+v=\left(c_{t_{x}}^{f-1}+v_{x},c_{t_{y}}^{f-1}+v_{y}\right),$$

where $v=(v_{x},v_{y})$ is the optical flow at $c_{t}^{f-1}$. Using optical-flow-based motion prediction, the location of a lost target is continuously updated. Consequently, if the target is occluded in one frame and reappears at a different location in subsequent frames, this motion prediction aids in estimating the likely location of the lost target. This approach improves the efficiency of reidentifying the lost target, leading to more accurate and reliable tracking performance.

Using Equation (2), we calculate the distance between each existing target and all detections $D^{f}$ and select only those targets within the distance threshold, $T_{e}$, to prioritize nearby detections. The network then encodes the feature vectors of all the possible pairings between the targets and the respective neighboring detections into a tensor, termed the feature permutation matrix Φ∈N×N×(520×2). For clarity, the dimension of the tensor Φ is described as Width×Height×Depth, where the width represents the targets and the height represents the detections. The feature vector of each target is concatenated with the feature vector of each one of its neighboring detections and arranged in Φ along its depth dimension. For each image frame in the video sequence, the number of targets and detections will vary. To maintain consistency in the tensor dimension, we introduce additional zero vectors into the matrix, ensuring that the size consistently remains at N×N×1040. The value chosen for *N* limits the maximum number of object detections in each frame, and through our analysis, N=80 was found to be a generous bound for the MOT benchmark datasets.

#### 3.1.2. Deep Feature Association Network

The objective of this component in the proposed MOT framework is to estimate the affinities between the selected detection–target pairs using the extracted feature vectors. This sub-network maps the tensor $\Phi \in R^{N\times N\times 1040}$ into a feature association matrix $A_{F}\in R^{N\times N}$. In the association matrix $A_{F}$, the columns account for the detections in the current frame and rows account for the active targets, both tracked and lost, from the previous trajectory. Besides, the scalar score in the matrix $A_{F_{\left(i,j\right)}}$ indicates the confidence of the $j^{th}$ detection and $i^{th}$ target ($d_{j}^{f}$ and ${TL}_{i}^{f-1}$) associated with the same identity.

We refer to the major component of this module as the deep compression network due to its functionality. The architecture of the deep compression network is inspired by the work presented in [30]. The input to this network is the tensor $\Phi \in R^{N\times N\times 1040}$, which accumulates the feature vectors of target–detection pairs. The output is an association matrix $A_{F}\in R^{N\times N}$ that encodes the similarity scores of these pairs. The specifications of the deep compression network architecture are detailed in Table 1. This network employs a five-layer convolutional neural network with 1×1 kernels for the task. As the tensor Φ passes through the network, it undergoes gradual dimension reduction along the depth dimension via the 1×1 kernels. These convolutional kernels enable the computation of similarity scores for each object pair without interference from neighboring objects.

**Figure 2:**
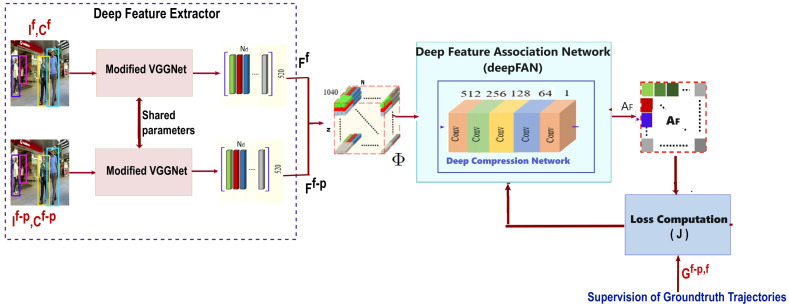
Approach to train the deep feature association network (deepFAN): Though an online MOT framework, the feature extractor is deployed as a single-stream model, and during training, it is considered as a two-stream network with shared parameters. The inputs to the feature extractor are the those two frames ($I^{f}$ and $I^{f-p}$, which are p frames apart, meaning they need not be adjacent frames), and we need to find the association between the detections and the centers of the object detections ($C^{f}$ and $C^{f-p}$ for $I^{f}$ and $I^{f-p}$). Since the input frames are non-adjacent, neighborhood detection estimation (NDE) is not valid and is not applied in the training pipeline. With the supervision of ground truth $G^{f-p,f}$, the cost function J is computed, and the weights of the deep compression network in deepFAN are updated.

***Training deep compression network:*** During the training process, the deep compression network learns the association function, which estimates the feature association matrix $A_{F}\in R^{N\times N}$ from the tensor $\Phi \in R^{N\times N\times 1040}$ for reliable online multiple object tracking. The approach used to train the compression network is illustrated in Figure 2. When we employ the proposed MOT framework (Figure 1) for online tracking, the feature extractor functions as a single-stream model. Additionally, during the tracking process, the input frames are presented in the order of the original video. We develop a specialized training strategy for the deep compression network, which enables the network to effectively learn the data association function by utilizing non-consecutive frame pairs from the video sequence. As a result, the network learns to reliably associate objects in a given frame with those in multiple previous frames, benefiting the framework by reducing identity switches and fragmented target trajectories. As shown in Figure 2, during training, we configured the network as a two-stream network of modified VGGNet with shared parameters. The feature extractor receives two frames, $I^{f}$ and $I^{f-p}$, separated by *p* frames (i.e., not adjacent frames), as well as the centers of object detection, $C^{f}$ and $C^{f-p}$, of pre-detected objects within those frames. These frame pairs are processed by modified VGGNets, which extract a 520-dimensional feature vector for each object detection in the input frames. We obtain feature matrices, $F_{D}^{f}$ and $F_{D}^{f-p}$, which accumulate the feature vectors for detections in each input frame $I^{f}$ and $I^{f-p}$. Since the input frames are non-adjacent, neighborhood detection estimation (NDE) is not applicable and is excluded from the training pipeline. The network arranges the columns of $F^{f}$ and $F^{f-p}$ to concatenate the columns of the two feature matrices along the depth dimension of the tensor $\Phi \in R^{N\times N\times 1040}$ in all possible permutations. To maintain consistency in the tensor dimensions, additional zero vectors are introduced, ensuring that the size remains N×N×1040. This tensor is then forward-passed through the compression network, which utilizes five convolutional layers with 1×1 kernels to map and estimate the feature association matrix $A_{F}\in R^{N\times N}$. For computing the error of the network during the learning process, we define a loss function J with the help of ground truth trajectories. A ground truth target association matrix $G\in R^{N\times N}$ is constructed as a binary matrix encoding the correspondence between the objects detected in frames $I^{f-p}$ and $I^{f}$. If the $i^{th}$ target in $I^{f-p}$ corresponds to the $j^{th}$ target in $I^{f}$, then the entry to the matrix $G_{\left(i,j\right)}^{f-p,f}$ is non-zero; otherwise, it is zero. The ground truth target association matrix G is subsequently compared with the network-predicted feature association matrix $A_{F}$, for the loss computation. The loss function of our training network is defined as

(4) $$J\left(G,A_{F}\right)=\frac{\sum_{i,j=1:N}G⊙-\left(\log A_{F}\right)}{\sum_{i,j=1:N}G},$$

where the symbol ⊙ represents the Hadamard product. The log operation on $A_{F}$ is performed elementwise, and $\sum_{i,j=1:N}$ finds the sum of all elements in the Hadamard product matrix. In the loss function, instead of computing the distance metric between the predicted association matrix $A_{F}$ and ground truth association matrix G, the probabilities encoded by the relevant coefficients of $A_{F}$ are maximized. During learning, the parameters of the compression network are updated by minimizing the loss over the training samples. The trained compression network is employed in online multiple object tracking.

Referring to Section 3.1.1, for consistency, additional zero vectors are introduced in the tensor Φ, so that the size will always be N×N×(520×2). Therefore, in the association matrix $A_{F}$$\in R^{N\times N}$, there are irrelevant values corresponding to the appended zero vectors. To reduce the irrelevant information and to normalize the matrix, we performed the following three post-processing steps over the feature association matrix $A_{F}$:

***(i)***  Truncation: Since we have only $N_{d}$ detections and $N_{TL}$ active targets, the  matrix $A_{F}\in R^{N\times N}$ is resized by truncating the matrix to $N_{TL}\times N_{d}$.
***(ii)***  RowwiseSoftmax: This operation normalizes the rows of the association matrix by fitting a separate probability distribution. The output row values are between the range [0, 1], and the total sums up to 1. Thus, each row of the resulting association matrix encodes the association probability between each active target in $I^{f-1}$ and all the detections in $I^{f}$.
***(iii)***  Thresholding: The association matrix values indicate the similarity between the detection and target objects. For a reliable data association, the values above the threshold $T_{a}$ are retained, and all other values below the threshold are set to zero.

These post-processing steps obtained for us an updated feature association matrix $A_{F}\in N_{TL}\times N_{d}$, which was further passed to the SSIM for the association update.

#### 3.1.3. Structural Similarity Index Metric for Association Update

The ultimate aim of the data association module is to develop a robust association model that delivers the most relevant information for achieving accurate multiple object tracking (MOT) performance. In the association matrix, a non-zero association value indicates a potential match between the corresponding target–detection pair. Traditionally, the detection with the highest association score is linked to the target trajectory. However, when multiple detections have similar or nearly equivalent association scores, uncertainties arise, leading to unreliable associations between detections and targets.

To address this issue, our proposed method incorporates the Structural Similarity Index Metric (SSIM) [11,12,13]. The SSIM is a widely recognized perceptual metric that measures the similarity between two images by leveraging their structural characteristics. By integrating the SSIM, we enhance the decision-making process for target associations. The proposed MOT framework considers the association results derived from the SSIM to make the final decision on the target association. This metric evaluates the effective similarity between the target and detection pairs, thereby improving the accuracy and reliability of the associations. We reduced the chance of wrong associations, which can happen when multiple detections have association scores that are very close to each other by using the SSIM. This makes sure that the detections are more accurately aligned with their targets.

Let ${\left(TL\right)}_{i}$ be the $i^{th}$ active target and ${\left\{d_{k}\right\}}_{k=1}^{K}$ be the detections corresponding to the non-zero association scores with the $i^{th}$ target. Also, let $d_{max}$ represent the detection with the highest score and $A_{F_{\left(i,max\right)}}$ be the highest score. As stated before, if there are other detections with similar or closer scores to this highest association score $A_{F_{\left(i,max\right)}}$, uncertainties occur in the target association. For the target ${\left(TL\right)}_{i}$, first, the set of detections with uncertainty $D_{s}$ is estimated as follows.

(5) $$\begin{array}{c} D_{s_{i}}=\left\{\forall d_{k}\;\mathrm{with}\;A_{F_{\left(i,k\right)}}\geq \left(A_{F_{\left(i,max\right)}}-0.1\right)\right\},\\ D_{s}={\left\{D_{s_{i}}\right\}}_{i=1}^{N_{TL}}. \end{array}$$

If the association matrix $A_{F}$ contains any zero rows, then the corresponding detection set in $D_{s}$ becomes an empty set. The SSIM module calculates the similarity score between the target and each detection in $D_{s}$. The output of the SSIM module is another SSIM association matrix $A_{S}\in R^{N_{TL}\times N_{d}}$ in which rows and columns represent the same active targets and detections as in $A_{F}$, but the entries replace the SSIM score of each valid pair, i.e.,

(6) $$A_{S_{\left(i,j\right)}}=\left\{\begin{array}{cc} SSIM(TL_{i},d_{j}), & \mathrm{if}\;d_{j}\in D_{s_{i}}\\ 0, & \mathrm{otherwise} \end{array}\right.$$

The SSIM-based association matrix, $A_{s}$, is utilized alongside $A_{F}$ to establish the final track association, A. The track association matrix is the result of adding both matrices $A_{F}$ and $A_{s}$ together.

### 3.2. Track Association

In a multiple object tracking scenario, an object detected in a video sequence has to undergo different state transitions. When the object detector detects the object for the first time, a new track is initialized in the trajectory list. Now, the object is in the tracked state and remains in the same state until re-detected in the subsequent frames. When the object gets occluded or goes out of the camera’s field of view, the object is transferred to the lost state. If the lost target re-appears, then the state is updated as tracked, and the tracking process resumes. The trajectory of the lost target is terminated if it stays long in the lost state. The data association algorithm in MOT helps to find the state of each detection in the video sequence. It estimates the correspondence between the object detections in the current frame and existing targets.

After accomplishing the training of the deep compression network with MOT datasets, we employed the trained network in the proposed MOT framework. Algorithm 1 summarizes the online tracking process in the proposed method. The objective of the MOT problem is to find the trajectory of all the possible targets present in the given input image sequence. Here, the MOT framework expects the present image frame $I^{f}$ and the object detection centers $C_{D}^{f}$ as its inputs. The detection feature matrix $F_{D}^{f}$ computed by the VGGNet feature extractor along with the target feature vector matrix $F_{TL}^{f-1}$ are utilized to create the feature permutation tensor Φ by a concatenation operation. We stored the feature vectors of the active targets, both tracked and lost targets, from the previous frame to find the association in the current frames. The tensor Φ forward-passed through the compression network is mapped to the association matrix $A_{F}$ as described in Section 3.1.2. Along with $A_{F}$, the SSIM-based association matrix $A_{s}$ is also utilized for finding the final track association, A. The track association method adapted in our framework is performed as follows.
**Algorithm 1:** Online multiple object tracking.**Input:** Video sequence, $V=\{I^{f}|f=1,2,\cdots ,F\}$ and object detections $D^{f}$
**Output:** Set of object trajectories, $T=\{\tau_{i}{\}}_{i=1}^{N}$,
1:**Initialization**: T←∅2:**for** Video frame $I^{f}$ in *V* **do**3:   **Feature extraction**4:   Input:$I^{f}$ and $C_{D}^{f}$5:   Output:$F_{D}^{f}\in R^{520\times N_{d}}$6:   **if** (f==1) **then**7:     Initialize trajectory $\tau_{i}^{1}$ for each detection,8:     state==tracked;9:   **else**10:     **Neighborhood estimation detection**11:     Input:$F_{D}^{f}$ and $F_{TL}^{f-1}$12:     Output: Tensor, $\Phi \in R^{N\times N\times 1040}$13:     **Feature association network**14:     Input:Φ15:     Output:$A_{F}$16:     **Structural Similarity Index Metric**17:     **for** each active target, ${\left(TL\right)}_{i}$, **do**18:        find $D_{s_{i}}$ detections with uncertainty.19:     **end for**20:     Input:${\left(TL\right)}_{i}$ and $D_{s_{i}}$, $i=1:N_{TL}$21:     Output:$A_{s}$22:     **Final track association matrix**23:     Input:$A_{F}$ and $A_{s}$24:     Output: Final track association matrix, $A=A_{F}+A_{s}$25:     **Target association**26:     Hungarian algorithm assigns detection to active targets.27:     Input:A28:     Output: trajectory, $\tau^{f}$29:     **if** tracked track $\tau_{j}^{\left(f-1\right)}$ not assigned to detection **then**30:        state == lost;31:     **end if**32:     **if** lost track $\tau_{j}^{\left(f-1\right)}$ assigned to detection $d_{m}$ **then**33:        state ==tracked;34:     **else**35:        state==inactive (if length of lost frames $>N_{inact}$, terminate track);36:     **end if**37:     **for** detections not covered by tracked and lost targets **do**38:        Initialize trajectory $\tau_{i}^{f}$.39:        state==tracked.40:     **end for**41:   **end if**42:**end for**.43:**return** trajectories of the objects, T.


In the first frame $I^{1}$, we initialize the trajectory list T with tracks $\{\tau_{i}{\}}_{i=1}^{N_{d}}$ by considering all the detections present in it as new tracked targets. Here, a track $\tau_{i}$ is an ordered set of the states of the $i^{th}$ target in the video sequence.

(7) $$\tau_{i}=\left\{s_{i}^{f_{e}},\cdots ,s_{i}^{f_{t}}\right\},\;s_{i}=\left(c_{x},c_{y},w,h\right)$$

In Equation (7), $f_{e}$ and $f_{t}$ are the entry and terminate frame for the $i^{th}$ target, $\left(c_{x},c_{y}\right)$ is the center of the target, and (w,h) are the width and height of the target. For each new target entry, the track is initialized with $\tau_{i}=s_{i}^{f_{e}}$. The trajectory list is updated after each input frame by employing the Hungarian algorithm [53] on the final association matrix A. In the track association part, the targets under the tracked state get higher priority. In this process, the targets that are associated with the detections are labeled as tracked, and the targets without association are labeled as lost. If the target stays in the lost state for a long time (say $N_{inact}$ as the length of frames; here, we chose the value as 20 frames), it is considered that the object has entered an inactive state, and we terminate the trajectory corresponding to that object. Finally, we initialize new trajectories for the detections that are not associated with the tracked targets.

## 4. Experiment Results and Discussion

In this section, we experimentally demonstrate the performance of the proposed deep MOT framework on the popular MOT benchmark datasets using the standard metrics. Here, we present the implementation details of our MOT framework, followed by the benchmark datasets and metrics used for performance analysis. We first conducted an ablation study on the validation dataset to understand the behavior of our approach better. Further, to obtain an authoritative reference when addressing MOT problems, the proposed framework was evaluated on the test datasets and the results compared with the state-of-the-art methods.

**MOT benchmark datasets:** The three popular MOT datasets, namely MOT15, MOT17, and MOT20 from the MOT Challenge [48,49], and UA-DETRAC [50,51] were employed here to test the performance of the proposed approach. These are the centralized benchmark datasets used to evaluate the tracking techniques in online multiple-object-tracking challenges. The annotated training video sequence, which includes the object detections and the ground truth labels in each frame, is used to train the models. The video sequences in the test datasets provided only object detections, whereas the ground truth labels remained unrevealed. Once the new MOT tracker has been submitted for performance analysis, the online MOT challenge hosting server evaluates the tracking results based on the standard MOT metrics [54].

### 4.1. Implementation Details

The proposed MOT framework was implemented in a Python framework, and the training was conducted on an NVIDIA Geforce Titan Xp 12GB GPU. We performed the training of the deep compression network on the MOT17 training dataset using the SGD optimizer. The hyperparameter values finally used in the training process were as follows: a batch size of 8, momentum of 0.9, an initial learning rate of 0.01, a weight decay of 0.0001, and the number of epochs per model of 120.

In the proposed MOT framework, the decision for the state transition of a target from lost to inactive is based on the hyperparameter $N_{inact}$, which is the maximum number of frames the target stays in the lost state before being transferred into an inactive state. In our analysis, we kept the value for $N_{inact}$ at 20. We chose N=80 as a generous bound for the MOT benchmark datasets, because it limits the maximum number of object detections in each frame. The feature extractor network has an input frame size of 900×900. Therefore, the network first resizes all the training and testing data to these dimensions before passing them through. The two threshold parameters used in this proposed framework are distance threshold, $T_{e}$ and association threshold $T_{a}$. The optimum value for the evaluation metrics obtained with the value of $T_{e}$ is equal to 0.35. In the thresholding step implemented as a post-processing part of the feature association matrix $A_{F}$, we used a association threshold $T_{a}$. For $T_{a}$ equal to 0.4, the proposed MOT framework obtained the optimum performance. The selection of $T_{e}$ and $T_{a}$ is explained in Section 4.2.

### 4.2. Ablation Study

In this section, to gain a deeper insight into the proposed MOT framework, we experimentally evaluated the contribution of different tracking components. Since the ground truth annotations are not provided for the MOT test datasets, the ablation study was conducted on the MOT15, MOT17, and MOT20 training datasets. We split the MOT training dataset into training and validation datasets. The splitting of the dataset is presented in Table 2. The proposed framework was trained on the training sequences, and the performance was evaluated on the validation sequences, as provided in Table 2.

#### Significance of the Proposed Tracking Components

This section follows the detailed analysis and discussion on the results obtained for the analyses of the three main components, (i) neighborhood estimation detection, (ii) feature association network, and (iii) SSIM association update. To investigate the significance of each component, we conducted several experiments by disabling one element at a time and studying the performance for the MOT metrics. Table 3 consolidates the evaluation results of the variants of the proposed method on all MOT evaluation metrics that demonstrate the significance of each module in the framework:

**(i)**  **Neighborhood detection estimation:**

**Figure 3:**
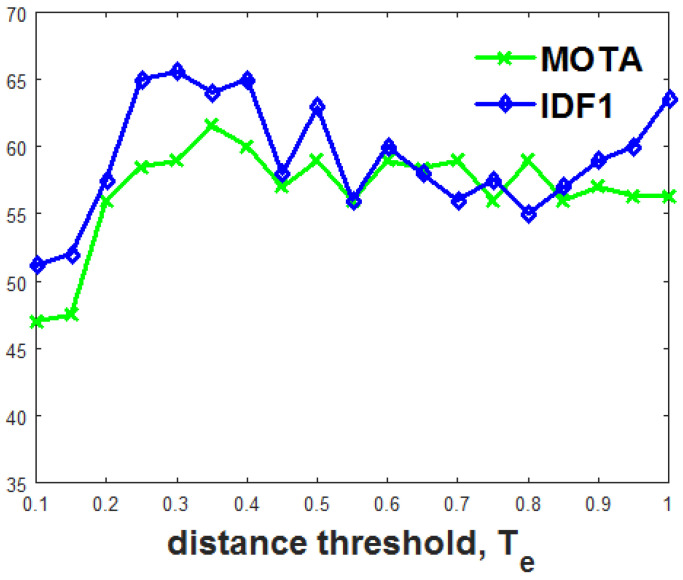
Performance analysis of proposed MOT framework with different values for NDE distance threshold, $T_{e}$. MOTA and IDF1 with different values for distance threshold $T_{e}$ on the MOT17 validation dataset are evaluated to find the optimum value of $T_{e}$.

As we discussed earlier, using NDE, we limited the search space for the association of the particular target into its neighborhood, assuming that the target will not move drastically from its position in a single frame change. The neighborhood of the target object was set to a limit using a distance threshold $T_{e}$. Figure 3 shows the MOTA and IDF1 with different values for distance threshold $T_{e}$. The optimum value for the evaluation metrics obtained with the value of $T_{e}$ is equal to 0.35, and we used this value of $T_{e}$ for the further experiments.

**Figure 4:**
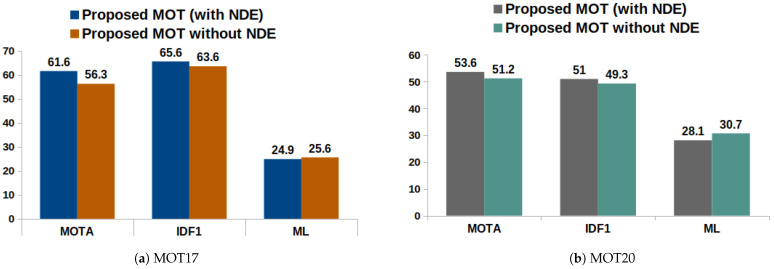
Analysis of neighborhood detection estimation on MOT17 and MOT20 validation sequences.

To demonstrate the significance of the proposed NDE in the MOT framework, we compared the performance of the trackers with and without NDE. Figure 4 shows three essential MOT metrics, MOTA, MOTP, and IDF1, evaluated on both the MOT17 and MOT20 validation datasets. Also, Table 3 tabulates the experimental results on all MOT metrics evaluated on the MOT17 and MOT20 validation datasets. The MOTA metric measures the overall accuracy of the detection and tracking, whereas the IDF1 scores highly depend on the association accuracy. The MOTP deals with the detection output. It is evident from the MOT scores that the scores improved with NDE. The MOTA is a metric derived from three types of detection–association errors: false positives, false negatives (missed targets), and identity switches. Since the NDE employed in the proposed method helps to choose only the reliable pairs for the association, it reduces the chance of wrong associations during the track estimation. It is clear from the results that NDE helps to reduce the wrong association, thereby reducing the identity switches, fragmentation, false negatives, and false positives, which in effect improves the MOTA. Also, the improvement in the IDF1 score also justifies that, with NDE, the association accuracy is improved.

**(ii)**  **Deep feature association network:**

**Figure 5:**
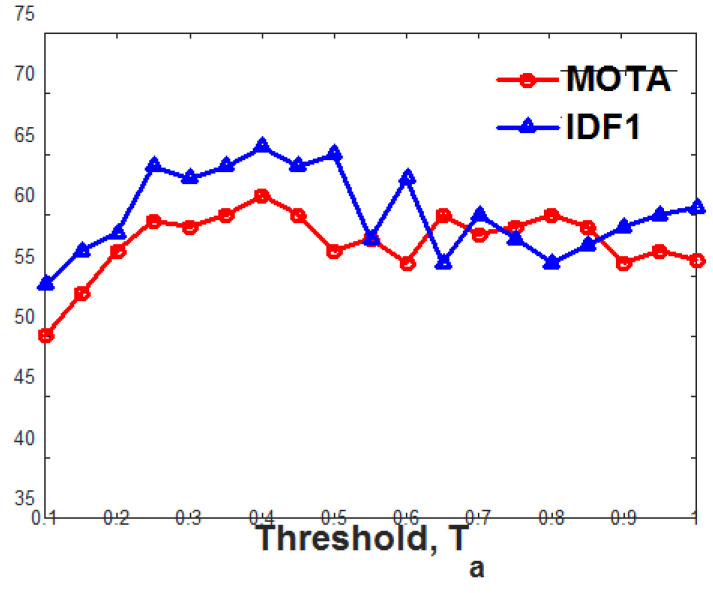
Performance analysis of proposed MOT framework with different values for threshold $T_{a}$. The MOTA and IDF1 with different values for threshold $T_{a}$ on the MOT17 validation dataset are evaluated to find the optimum value of $T_{a}$.

The deep feature association network (deepFAN) estimates the association matrix that encodes the association scores of each detection–target pair. The module includes three post-processing steps that remove the irrelevant information from the association matrix, improving the trajectory estimation. In the thresholding step, we used a hyperparameter, threshold $T_{a}$. Figure 5 plots the MOTA and IDF1 scores of the proposed MOT framework with different values of $T_{a}$, and an optimum result was obtained for $T_{a}$ equal to 0.4.

**Figure 6:**
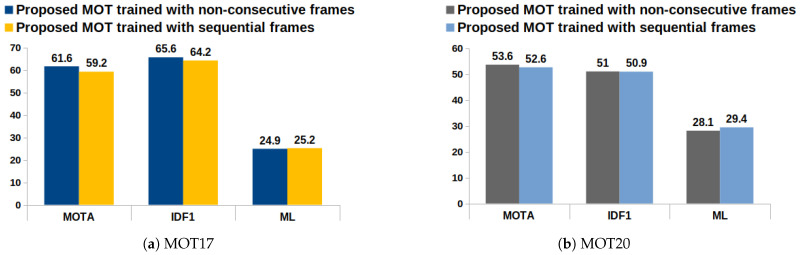
Analysis of deep feature association network on MOT17 and MOT20 validation sequences.

Figure 6 and Table 3 show the performance of the proposed training strategy on the MOT17 and MOT20 validation sequences in terms of the MOT metrics. The deep network was trained on the MOT dataset with the strategy that the input frames need not be sequential, i.e, non-consecutive input frames. Therefore, the data association model becomes robust to the tracking challenges such as appearance variation, illumination changes, scale changes, etc. It also helps in the re-identification of the lost targets and handles object occlusions, thereby reducing the identity switches and fragmentation issues in MOT. The experimental results showed that it improves the overall MOT performance.

**(iii)**  **SSIM association update:**

**Figure 7:**
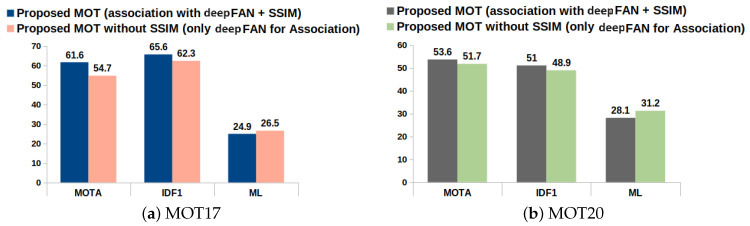
Analysis of Structural Similarity Index Metric on MOT17 and MOT20 validation sequences.

The SSIM introduced in the proposed model can be considered as a second opinion when an ambiguity in association occurs. Figure 7 and Table 3 show the importance of SSIM association by evaluating the model on MOT metrics with the MOT17 and MOT20 validation data sequences. As the performance of the data association algorithm improves, we will obtain better association estimation, which will enhance the tracker’s tracking performance. It is observed from the results that the SSIM enhances the performance of the data association algorithm. It reduces the false negatives and identity switches and, hence, the MOTA. Also, the high IDF1 score validates the significance of SSIM association in the refinement of the association matrix.

### 4.3. MOT Benchmark Evaluation

This section shows the experimental evaluation of the proposed method on the benchmark datasets. Table 4 summarizes and compares our results with state-of-the-art algorithms on MOT benchmark datasets and Table 5 on UA-DETRAC. Here, we also show the effects of systematically adding neighborhood detection estimation, non-sequential training, and SSIM association update in the proposed tracker.

The benchmark evaluation results show that the proposed MOT framework performs very well in terms of the MOT evaluation metrics. We would like to emphasize that the metric scores for identity switches, fragmentation, and false negatives are reduced, indicating the reduction in the wrong association among detection target pairs. This results in better accuracy (MOTA). Also, the IDF1 score is improved, which is a clear indication of the association accuracy. This shows the robustness and efficiency of the proposed data association method.

We compared our results with recent state-of-the-art methods. The benchmark evaluation result depicts that the proposed data association method outperforms the state-of-the-art DAN model [30]. In particular, the nearest neighborhood estimation employed for detection–target feature pair selection reduces the association mismatch and improves the computational efficiency. The post-processing steps after deepFAN also help enhance the association accuracy and reduce the computational complexity. Here, the employment of the SSIM reduces the ambiguity in the data association.

The tracking results of the proposed tracker with the UA-DETRAC dataset are summarized in Table 5. Here, we opted for the EB detector [63] for a fair comparison. Since the trackers in Table 5 used different detectors, the name of the tracker is given along with the detector used. The proposed method gives better results on the UA-DETRAC evaluation compared with other approaches and can also be effectively used for vehicle tracking.

## 5. Conclusions

Developing a better data association framework is very crucial for robust multiple object tracking. This research work proposes two important contributions to enhance the data association. The first one is by introducing neighborhood detection estimation (NDE) only to retain reliable detection–target pairs. Secondly, the SSIM association component is proposed to reject ambiguous associations with high or near high association scores. A comprehensive evaluation strategy was adopted to understand and study the impacts of our technical contributions on popular multiple object tracking benchmarks. Further, we carried out a systematic ablation study to pinpoint the benefits of each proposal. We compared our proposals with recent multiple object tracking frameworks. Our studies found that the proposed tracker gave very low identity switches, which is one of the crucial factors in ranking various trackers. Further, the proposed tracker also achieved very high overall MOTA and IDF1 scores. Another factor that we wish to highlight here is that the proposed framework rejects ambiguous associations and employs only the neighboring detections for data associations. Ultimately, this leads to achieving higher tracking speed, which is another important factor in multiple object tracking. In the future, we would like to deploy this tracker in real-time tracking scenarios by augmenting a dedicated object-detection module along with the proposed tracker for real-world applications.

## Figures and Tables

**Table 1:** Architectural details of the deep compression network. Here, we use stride = 1 and ReLU activation in each layer. BN indicates batch normalization, and Y/N denotes whether B is applied or not.

Index	Input Channels	Output Channels	BN
0	1040	512	Y
3	512	256	Y
6	256	128	Y
9	128	64	N
11	64	1	N

**Table 2:** Training and validation data sequences for ablation study of proposed MOT framework on the MOT17 and MOT20 benchmark.

Training	Validation
**MOT15**	
TUD-Stadtmitte	TUD-Campus
ETH-Bahnhof	ETH-Sunnyday, ETH-Pedcross2
ADL-Rundle-6	ADL-Rundle-8, Venice-2
KITTI-13	KITTI-17
**MOT 17**	
MOT17-02	MOT17-04
MOT17-05	MOT17-09
MOT17-10	MOT17-11, MOT17-13
**MOT 20**	
MOT20-01	MOT20-02
MOT20-03	MOT20-05

**Table 3:** Analysis of the proposed framework on the MOT validation datasets and comparison with different proposed tracker variants by disabling different components. (The best values are in **boldface**. ↑ indicates that a higher value is better, and ↓ indicates a lower value is better.)

Tracker	MOTA↑	IDF1↑	MT↑	ML↓	FP↓	FN↓	Recall↑	Precision↑	FAF↓	IDSw↓	Frag↓
**MOT20**											
Proposed MOT	**53.6**	**51.0**	**31.6**	**28.1**	**12,094**	**135,548**	**55.4**	**85.5**	**1.2**	**1264**	**1853**
Proposed MOT without NDE	51.2	49.3	29.6	30.7	14,296	154,780	52.0	79.3	**1.2**	1573	2098
Proposed MOT trained with sequential frames	52.6	50.9	30.9	29.4	12,944	139,547	55.3	95.6	1.5	1463	1921
Proposed MOT without SSIM	51.7	48.9	27.3	31.2	14,991	141,703	53.1	97.4	2.1	2234	3084
**MOT17**											
Proposed MOT	**61.6**	**65.6**	**34.4**	**24.9**	**8361**	**76,123**	**62.1**	**88.8**	**1.1**	**695**	**969**
Proposed MOT without NDE	56.3	63.6	33.2	25.6	9662	86,425	58.7	85.7	1.2	834	1167
Proposed MOT trained with sequential frames	59.2	64.2	33.4	25.2	8691	77,453	59.3	87.4	**1.1**	726	1098
Proposed MOT without SSIM	54.7	62.3	32.4	26.5	10,231	89,653	61.5	88.4	1.5	832	1217
**MOT15**											
Proposed MOT	**46.2**	**62.3**	**22.2**	**15.4**	**2134**	**10,162**	**55.4**	**85.5**	**0.8**	**121**	**286**
Proposed MOT without NDE	38.2	71.9	18.0	20.0	3094	10,943	52.0	79.3	1.0	236	341
Proposed MOT trained with sequential frames	43.2	74.2	21.1	16.4	2652	10,295	54.8	82.4	1.0	134	291
Proposed MOT without SSIM	38.1	73.1	19.2	21.8	2851	11,343	53.6	82.1	1.1	216	312

**Table 4:** Comparison of the proposed MOT framework on the MOT test dataset with state-of-the-art trackers. (Red for the best values and blue for second place. NA represents the values that are not available in the publications. ↑ indicates that a higher value is better, and ↓ indicates a lower value is better.)

Tracker	MOTA↑	IDF1↑	MT↑	ML↓	FP↓	FN↓	Recall↑	Precision↑	FAF↓	IDSw↓	Frag↓
**MOT20**											
**Proposed MOT**	58.9	59.7	30.7	19.2	31,063	158,876	61.7	89.2	4.5	1842	3126
Proposed MOT without NDE	55.4	57.9	29.2	20.7	33,473	161,875	60.5	88.1	5.6	2187	4125
Proposed MOT trained with sequential frames	57.5	58.6	30.9	19.8	31,974	159,174	58.6	88.5	4.9	1925	3215
Proposed MOT without SSIM	56.1	57.6	28.7	20.1	33,542	168,654	67.9	87.7	5.3	2213	3982
MPN Track [55]	57.6	59.1	38.2	22.5	16,953	201,384	61.1	94.9	3.8	1210	1420
TMOH [56]	60.1	61.2	46.7	17.8	38,043	165,899	67.8	90.2	8.5	2342	4326
MPTC [57]	60.6	59.7	51.1	16.7	45,318	153,978	70.2	88.9	10.1	4533	5163
TBC [58]	54.5	50.1	33.4	19.7	37,937	195,242	62.3	89.5	8.5	2449	2580
**MOT17**											
**Proposed MOT**	58.6	60.8	24.1	29.1	20,230	212,345	59.5	93.9	0.8	1122	1943
Proposed MOT without NDE	56.4	58.3	22.6	31.9	21,237	222,746	54.0	92.5	0.9	1782	2153
Proposed MOT trained with sequential frames	57.8	60.1	23.5	29.9	20,934	215,145	57.5	93.3	0.8	1352	1986
Proposed MOT without SSIM	54.9	52.8	20.9	31.1	22,237	229,447	55.9	92.9	1.1	2668	3469
DAN [30]	52.4	49.5	21.4	30.7	25,423	234,592	58.4	76.9	NA	8431	14,797
Tractor++ [59]	53.5	52.3	49.5	36.6	12,201	248,047	56.0	96.3	0.7	2072	4611
DMAN [44]	48.2	55.7	19.3	38.3	26,218	263,608	53.3	92.0	1.5	2194	5378
DEEP TAMA [60]	50.3	53.5	19.2	37.5	25,479	252,996	55.2	92.4	1.4	2192	3978
FAMNet [42]	52.0	48.7	18.1	33.4	14,138	253,616	55.1	95.6	0.8	3072	5318
TT17 [61]	54.9	63.1	24.4	38.1	20,236	233,295	58.7	94.2	1.1	1088	2392
**MOT15**											
Proposed MOT	52.5	60.0	33.8	25.8	6837	21,218	64.8	85.3	1.2	370	784
Proposed MOT without NDE	49.4	58.8	29.4	28.2	10,774	23,204	64.1	84.6	1.2	628	1090
Proposed MOT trained with sequential frames	51.1	59.0	31.1	27.6	8070	21,292	64.6	84.7	1.7	677	922
Proposed MOT without SSIM	47.5	47.8	23.4	26.4	9531	25,502	58.5	83.7	1.3	1040	1350
MPN Track [55]	51.5	58.6	31.2	25.9	7620	21,780	64.6	83.9	1.3	375	872
Tracker++ [59]	46.6	47.6	18.2	27.9	4624	26,896	56.2	88.2	0.8	1290	1702
GNN Match [62]	46.7	43.2	21.8	28.2	6643	25,311	58.8	84.5	1.1	820	1371

**Table 5:** Comparison of the proposed MOT framework on the test dataset, UA-DETRAC, with state-of-the-art trackers. (Red for the best values and blue for second place. NA represents the values that are not available in the publications.)

UA-DETRAC									
**Tracker**	**PR-MOTA↑**	**PR-MOTP↑**	**PR-MT↑**	**B**	**PR-FP**↓	**PR-FN↓**	**PR-IDSw↓**	**B**↓	**Hz**
**EB [63] + Proposed MOT**	23.4	30.9	17.5	16.8	8253.6	17,532.6	462.2	721.1	12.7
EB + Proposed MOT without NDE	21.1	28.5	16.5	17.9	9757.5	19,572.9	537.9	794.5	10.9
EB + Proposed MOT trained with sequential frames	21.7	29.1	16.9	18.1	9034.2	18,854.5	489.1	774.1	12.1
EB + Proposed MOT without SSIM	20.8	28.1	16.0	18.7	10,054.3	20,834.6	549.7	875.4	14.5
EB + DAN [30]	20.2	26.3	14.5	18.1	9747.8	135,978.1	518.2	NA	6.3
compACT [64] + FAMNet [42]	19.8	36.7	17.1	18.2	14,988.6	164,432.6	617.4	970.2	NA
EB + IOUT [65]	19.4	28.9	17.7	18.4	14,796.5	171,806.8	2311.3	2445.9	NA
R-CNN [7] + IOUT	16.0	38.3	13.8	20.7	22,535.1	193,041.9	5029.4	5795.7	NA
compACT + CMOT [66]	12.6	36.1	16.1	18.6	57,885.9	167,110.8	285.3	1516.8	3.8

## Data Availability

The source code has been uploaded to GitHub, which can be accessed at the following link: “https://github.com/aswathyiist123/MOT_deepFAN_SSIM (accessed on 1 July 2024)”.
